# Unveiling the Rarity: A Case Report of Malignant Melanoma of the Rectum

**DOI:** 10.7759/cureus.62747

**Published:** 2024-06-20

**Authors:** Nikhil Thatipalli, Darshana Tote, Anup Zade, Tushar Dahmiwal, Suhit Naseri

**Affiliations:** 1 General Surgery, Jawaharlal Nehru Medical College, Datta Meghe Institute of Higher Education & Research, Wardha, IND; 2 General Surgery, Mahatma Gandhi Institute of Medical Sciences, Wardha, IND; 3 Pathology, Jawaharlal Nehru Medical College, Datta Meghe Institute of Higher Education & Research, Wardha, IND

**Keywords:** chemotherapy, abdomino-parineal resection, anorectal junction, rectal bleeding, colon, malignant melanoma

## Abstract

Malignant melanoma of the rectum is an aggressive malignant tumor with anal pain and rectal bleeding as common clinical symptoms with a low incidence. Intestinal metastases are a common form of cutaneous melanoma. On a cellular level, the fibrous stroma is observed to be in the form of compact nests with a signet ring-like appearance. This is a case of a 67-year-old male with major complaints of altered bowel habits, a history of rectal bleeding for four months, and pain during defecation. Upon digital rectal examination, nearly half of the anal lumen was occupied by a fleshy mass. A detailed examination showed an ulcerating, black-colored nodule extending from the anorectal junction. Imaging studies confirmed a polypoidal lesion in the distal rectum. Histopathological examination of the biopsies revealed features consistent with malignant melanoma, supported by positive staining for HMB-45 and S-100 markers. The patient underwent an open abdominoperineal resection, followed by postoperative management and the initiation of chemotherapy. This case can be noted as underscoring the criticality of the diagnosis and treatment of rectal malignant melanoma and highlighting the importance of early recognition for improved patient outcomes.

## Introduction

Rectal malignant melanomas are uncommon and highly aggressive, affecting the rectal region of the digestive tract. It constitutes a minute fraction of all melanomas diagnosed worldwide, comprising less than 1% of cases, and represents only a small percentage, ranging from 0.5% to 4% of anorectal malignancies. This rarity contributes to the challenges of its diagnosis and treatment. The demographic profile of individuals affected by rectal malignant melanoma typically skews toward females, and symptoms often manifest during the fifth or sixth decade of life [1,2]. Moreover, studies have shown a strong association between Caucasian ethnicity and the incidence of malignant melanoma. For instance, Caucasians have been reported to have a significantly higher risk, with a 20-fold increase in the incidence of cutaneous melanoma compared to African Americans. This racial disparity underscores the importance of considering demographic factors in understanding disease prevalence and risk assessment [3,4]. One of the notable difficulties in diagnosing rectal malignant melanoma lies in the nonspecific nature of its presenting symptoms. Patients often report general complaints such as anal pain or rectal bleeding, which can easily be attributed to other more common conditions. Furthermore, diagnosing anal melanoma is particularly challenging due to the lack of visible pigmentation in a significant proportion of lesions. Up to 80% of cases may present without visible pigmentation, and approximately 20% of tumors may lack melanin histologically, further complicating the accurate diagnosis [5,6]. The prognosis for patients with rectal malignant melanoma is notably grim, with a five-year survival rate ranging from 10% to 15% and a median survival of only 24 months. These poor outcomes can be attributed to the aggressive nature of these tumors and effective treatment outcomes. Additionally, the lack of consensus on the most effective surgical approach further adds to the complexity of managing this condition. Anorectal malignant melanomas have a propensity to spread along submucosal planes, often making them difficult to resect completely at the time of diagnosis. Furthermore, metastases are responsible for the majority of patient deaths, highlighting the urgent need for more effective treatment strategies [1,5-8]. A major challenge for the diagnosis and treatment of rectal malignant melanoma lies in its rarity, nonspecific symptoms, and aggressive behavior. Addressing these challenges requires a comprehensive understanding of the disease’s epidemiology, clinical presentation, and treatment options, underscoring the importance of continued research and multidisciplinary collaboration in improving patient outcomes.

## Case presentation

**Figure 1:**
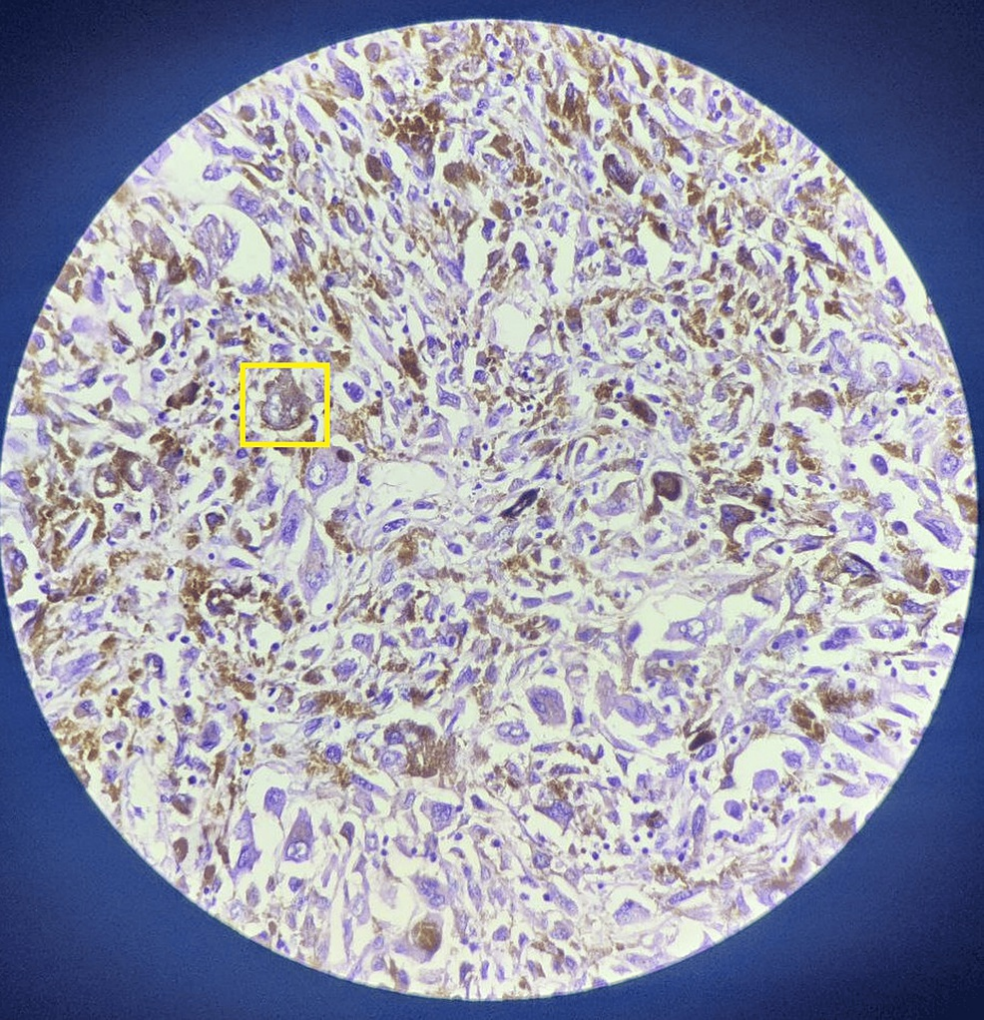
H&E staining at 40x magnification The highlighted area shows multinucleated enlarged cells with conspicuous pigment.

A 67-year-old male visited our hospital with major complaints of altered bowel habits, pain during defecation, and rectal bleeding for four months, with a history of weight loss of 10 kilograms over two months and loss of appetite. The patient did not have any smoking or tobacco addictions. On admission, he was hemodynamically stable, with hemoglobin at 8.5 mg/dl. His BMI was 16.6, which can also be marked as an indicator of overall poor health. Karnofsky Performance Status for the patient was scored between 60 and 70. The patient was examined for a thorough clinical examination, with an observation of a substantial fleshy mass occupying nearly half of the anal lumen noted by digital rectal examination. The rest of the assessments were found to be unremarkable. A colonoscopy revealed an ulcer-proliferative, friable mass in the lower region of the rectum, which partially obstructed the lumen and was spanning approximately 15 cm into the rectum from the anal verge. Multiple biopsies were taken from the growth. The biopsy specimen from the anorectal mass showed spindled and epithelioid cells, some of which were multinucleated. The cell nuclei were enlarged and prominent, with vesicular chromatin. The presence of conspicuous pigment within the tumor cells was noted (Figure 1).

**Figure 2:**
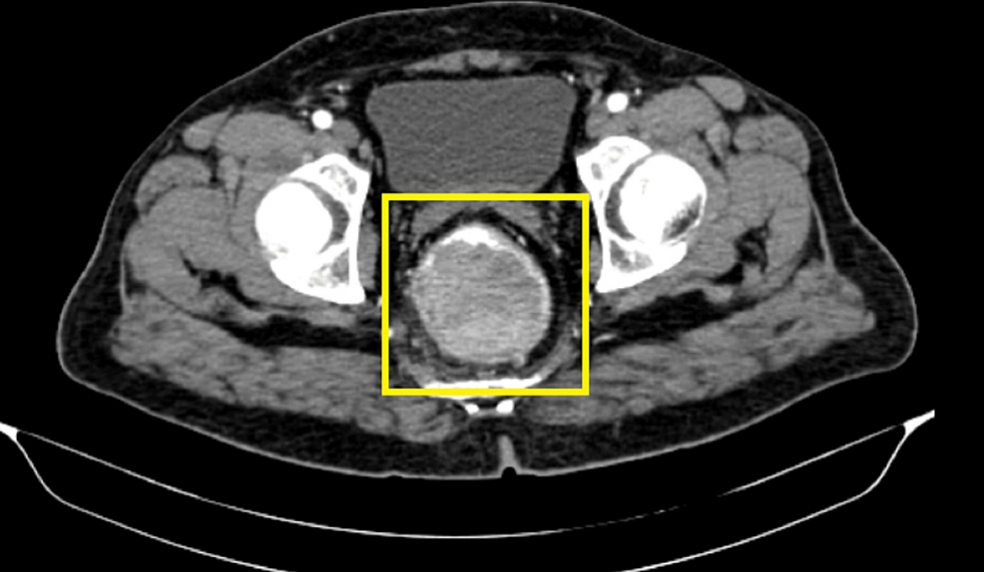
CT image showing a polypoidal lesion

**Figure 3:**
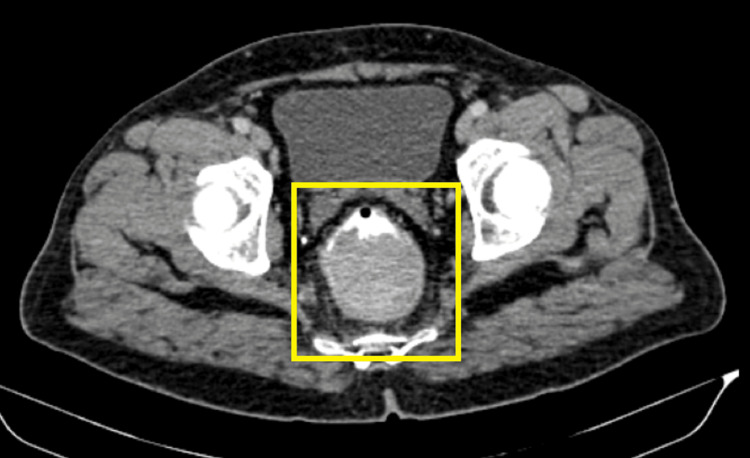
CT image showing a polypoidal lesion

Further radiological imaging of the abdomen and pelvis by CT revealed a heterogeneously enhancing polypoidal lesion emerging from the posterior wall of the distal rectum, measuring 7 × 6.5 × 6 cm, resulting in near-total obliteration of the lumen. Minimal fat stranding was noted in the mesorectal space, with intact fat planes adjacent to surrounding organs (Figure 2, Figure 3).

**Figure 4:**
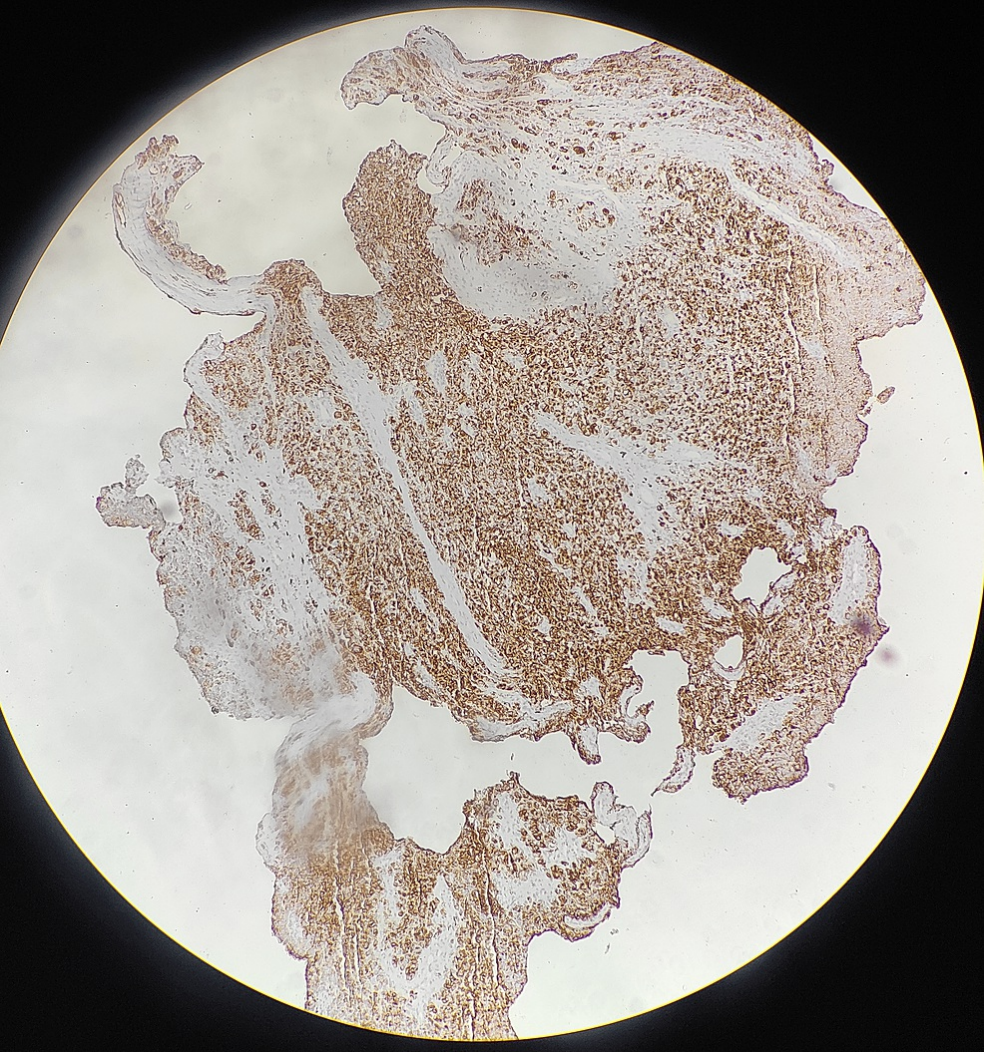
A positive HMB 45 staining indicative of malignant melanoma

The mass was analyzed histopathologically through multiple biopsy samples, which confirmed the presence of malignant melanoma of the colon with positive staining for S-100 and HMB-45 markers (Figure 4).

**Figure 5:**
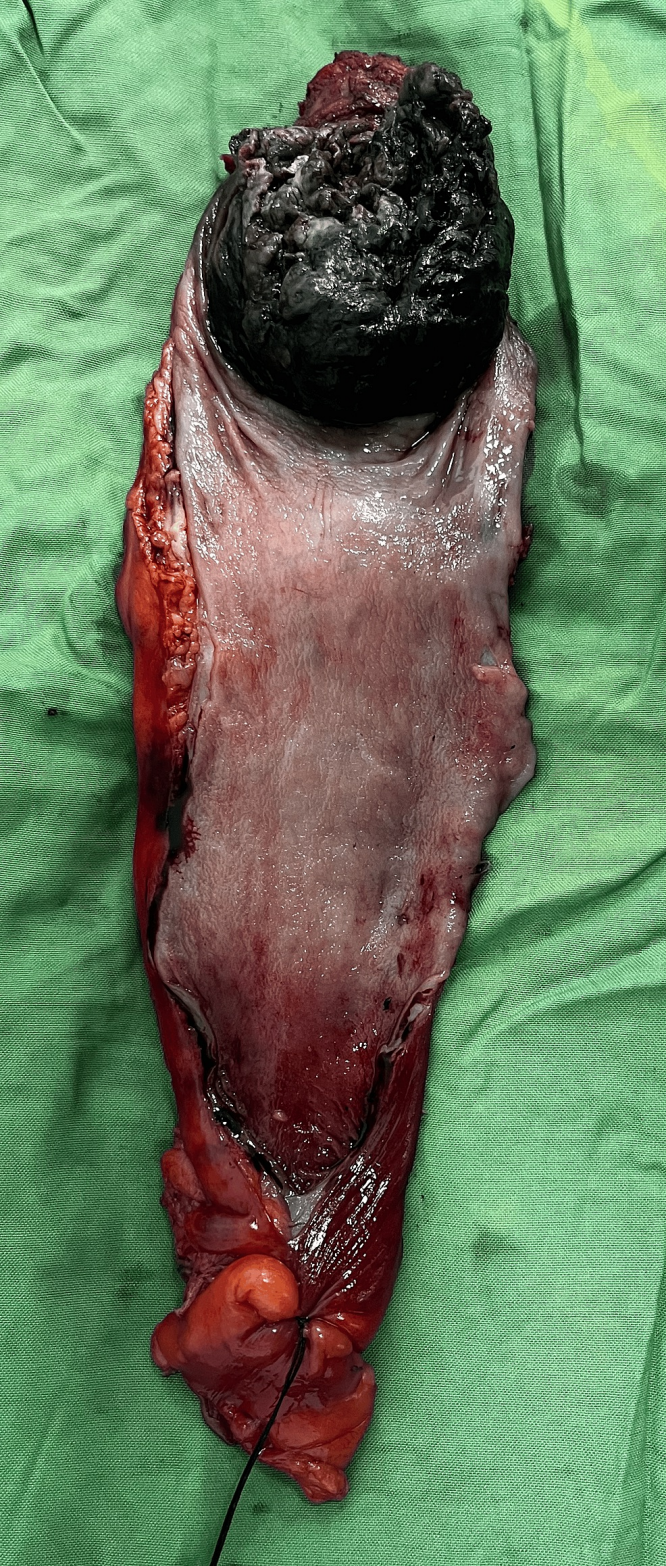
Excised specimen revealing the black-colored ulcerative friable mass

The patient underwent an open abdominoperineal resection (APR) as per the advice of a multidisciplinary tumor board. The surgical procedure involved a midline vertical incision, meticulous dissection of the peritoneum to create an avascular presacral plane anterior to the presacral nerves, and excision of the rectum, along with preservation of surrounding structures. The inferior mesenteric pedicle was doubly ligated and divided, just after the origin of the first sigmoid branch, followed by the removal of the sigmoid colon. Perineal incision and dissection were performed to extract the specimen, with subsequent closure of the perineal wound and the creation of an end sigmoid stoma. Postoperatively, the patient received intravenous fluids, analgesics, and antibiotics, achieving functional stoma by postoperative day 5. The specimen was sent for histopathological examination, which confirmed the sample to be malignant melanoma of the colon, confirming the diagnosis (Figure 5). The pathological staging of the tumor was pT1N0Mx, and three lymph nodes were identified on histopathology.

Sutures were removed sequentially from both abdominal and perineal incisions by postoperative day 13. The patient was advised to undergo follow-up examinations and radiological imaging.

## Discussion

Anorectal melanoma is an aggressive tumor with a propensity for lymphatic spread to the inferior mesenteric or inguinal nodal basins. Common sites of metastasis include inguinal, mesenteric, para-aortic, and hypogastric lymph nodes, the brain, skin, lung, and liver. Approximately 60% of cases present with locoregional lymph node metastases at initial diagnosis, while distant metastases are found in 26-38% of cases [1-4]. Melanocytes, originating from the embryological neural crest, are observed as precursor cells for both mucosal and cutaneous melanoma. These cells move in different regions of the body, primarily in the skin of the developing fetus, although they can also be found on mucosal surfaces and ocular structures such as the retina and uveal tract. As a result, cutaneous melanomas comprise the vast majority, accounting for over 90% of all cases of melanoma. Ocular melanoma represents approximately 5% of the remaining cases, with melanomas of unknown origin and mucosal melanomas comprising smaller percentages, at 2% and 1%, respectively [3,4]. Exposure of melanocytes to ultraviolet (UVB) light, a known carcinogenic stimulus, can trigger malignant transformation, particularly in cutaneous melanomas. Interestingly, anorectal melanoma does not appear to have a direct correlation with UVB exposure [9]. Instead, factors such as HIV and human papillomavirus infections have been implicated in the increased incidence of anorectal melanoma, suggesting a potential role for immunological mechanisms in its development [9,10]. Anorectal melanomas predominantly arise from melanocytes located in the squamous and anal transition zones of the rectum, with the dentate line serving as the origin for most of these tumors. Anal margins and anal canals have been reported to have approximately 65% of anorectal melanomas. The most common clinical presentations of these tumors are noted as palpable masses in the anorectal region, anal pain during defecation, bleeding, and altered bowel habits. Additional symptoms may include pruritus, tenesmus, prolapsed hemorrhoids, changes in fecal patterns, and diarrhea, with advanced stages exhibiting anemia, fatigue, and weight loss [4,9]. These anorectal lesions might be misdiagnosed as benign polyps or hemorrhoids [7,9]. Sigmoidoscopy is recommended for procuring the biopsy samples and analyzing the cause of the symptoms, along with endorectal ultrasound imaging in nodal assessment and surrounding tissues [9]. While CT imaging of the abdomen and pelvis can be helpful in disease evaluation, in cases of neoplasia, MRI and CT imaging can provide valuable insights into tumor extent and characteristics [3,7,9]. MRI, particularly T1-weighted imaging, reveals a high signal intensity in the melanotic component, aiding in the characterization of the lesion’s extraluminal extent [11,12]. Despite the challenges of detecting melanin in anorectal disease, its presence can be valuable in cases with a diagnostic challenge. Immunohistochemical markers such as vimentin, HMB-45, and S-100 are crucial in the final diagnosis and differentiating these melanomas from epidermoid carcinomas. Additionally, mutations in the KIT gene, associated with leukemia and gastrointestinal stromal tumors, have been linked to the pathogenesis of malignant melanoma [4,5]. Patients with anorectal melanoma have a five-year survival rate ranging between 16% and 34%, with a decrease in disease-free survival rate to 16% for those diagnosed with metastasis [4,9]. Unfortunately, treatment outcomes for anal melanoma remain suboptimal, with surgery recommended as a primary treatment modality, although there are different opinions on the optimal surgical approach, whether wide local excision (WLE) or APR should be followed. Recent studies suggest that WLE may offer comparable disease control while minimizing surgical morbidity [1,3,4,9]. However, lymph node dissection can be crucial where the disease is clinically evident or detected through sentinel lymph node techniques [2-4]. Systemic therapy for disseminated disease remains challenging due to the lack of established guidelines. Immune therapy, radiation therapy, and chemotherapy are limited in their efficacy. Commonly used drugs in adjuvant therapy include interleukins (IL-2), interferon B, dacarbazine, vinblastine, and cisplatin. Dacarbazine, in particular, has shown a partial response in approximately 20% of patients four to six months after initiating the treatment [2,8,9]. Research studies have found that the combination of WLE and adjuvant locoregional radiation therapy produced a similar disease control with less functional loss as compared to adjuvant partial radiotherapy [1]. The determination of optimal treatment strategies for anal melanoma can be attributed to its low incidence and rarity. Continued research efforts are crucial for improving the management and outcomes of this challenging disease [3,4,7,9].

## Conclusions

Malignant melanoma of the rectum is challenging to diagnose and manage due to its nonspecific symptoms and advanced stage at presentation, with surgical resection as the mainstay treatment, although the optimal approach is still under debate. Adjuvant therapies have shown limited efficacy, emphasizing the need for further research to establish standardized treatment protocols. Despite its rarity, early recognition and appropriate intervention are paramount for improving patient outcomes. Overall, this case underscores the importance of a high index of suspicion, timely diagnosis, and a multidisciplinary management approach for optimizing the prognosis of rectal malignant melanoma.
